# Immobilised-enzyme microreactors for the identification and synthesis of conjugated drug metabolites†

**DOI:** 10.1039/d3ra03742h

**Published:** 2023-09-18

**Authors:** Bradley Doyle, Leigh A. Madden, Nicole Pamme, Huw S. Jones

**Affiliations:** a School of Natural Sciences, University of Hull HU6 7RX UK; b Centre for Biomedicine, University of Hull HU6 7RX UK; c Department of Materials and Environmental Chemistry, Stockholm University 106 91 Stockholm Sweden; d Institute of Cancer Therapeutics, University of Bradford BD7 1DP UK H.S.Jones@bradford.ac.uk

## Abstract

The study of naturally circulating drug metabolites has been a focus of interest, since these metabolites may have different therapeutic and toxicological effects compared to the parent drug. The synthesis of metabolites outside of the human body is vital in order to conduct studies into the pharmacological activities of drugs and bioactive compounds. Current synthesis methods require significant purification and separation efforts or do not provide sufficient quantities for use in pharmacology experiments. Thus, there is a need for simple methods yielding high conversions whilst bypassing the requirement for a separation. Here we have developed and optimised flow chemistry methods in glass microfluidic reactors utilising surface-immobilised enzymes for sulfonation (SULT1a1) and glucuronidation (UGT1a1). Conversion occurs in flow, the precursor and co-factor are pumped through the device, react with the immobilised enzymes and the product is then simply collected at the outlet with no separation from a complex biological matrix required. Conversion only occurred when both the correct co-factor and enzyme were present within the microfluidic system. Yields of 0.97 ± 0.26 μg were obtained from the conversion of resorufin into resorufin sulfate over 2 h with the SULT1a1 enzyme and 0.47 μg of resorufin glucuronide over 4 h for UGT1a1. This was demonstrated to be significantly more than static test tube reactions at 0.22 μg (SULT1a1) and 0.19 μg (UGT1a1) over 4 h. With scaling out and parallelising, useable quantities of hundreds of micrograms for use in pharmacology studies can be synthesised simply.

## Abstract 2

On-chip continuous-flow synthesis of metabolites from glucuronidation and sulfonation reactions to enable synthesis of analytical standards and study drug metabolism.

## Graphical abstract

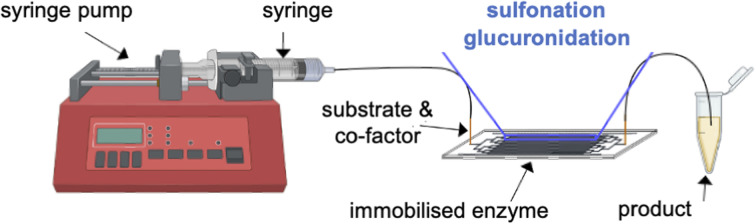

## Introduction

Chemicals entering the human body undergo significant oxidative, reductive and conjugative metabolic processing, which can drastically alter their pharmacokinetic and pharmacological activities.^1^ Currently, when assessing candidate compounds in drug discovery, tests are generally performed on the unmodified parent compound using *in vitro* systems with little consideration of these metabolic processes.^2^ It is only when testing moves to *in vivo* models that metabolic processes are considered.^3^ This is a major contributor to candidate compound failure and results in wasting of time, resources and ultimately delays in improving patient outcomes.^4^ Key reasons for this lack of early integration of compound metabolism into drug development are a lack of understanding of which metabolites are likely to be produced, and, significantly, the lack of convenient synthesis routes of the compounds likely to be formed during human metabolism.

The oxidative and conjugative metabolism of drugs significantly impacts on drug and xenobiotic absorption, distribution, and excretion at cellular, tissue and organism levels.^5,6^ These processes are predominantly enzymatically-driven, however some conjugation reactions, such as glutathione conjugation, can be spontaneous.^7^ The broad aim of these metabolic processes is to yield metabolites which are more easily excreted. As these processes involve oxidative, reductive and conjugative modifications to the parent drug structure, they result in significant changes to the physiochemical properties of the drugs, thus modifying drug pharmacology.^8^ In order to understand the pharmacology of a potential drug, including on-target, off-target and toxic effects, knowledge of the biological effects of metabolites in addition to those of the parent drug is essential in pharmacological research.

An additional challenge in human drug trials is that although prediction of likely metabolites is currently available, the lack of ability to produce sufficient amounts of these metabolites for use as analytical standards means that detecting and quantifying them with confidence is very challenging.^9^ This lack of ability to assess purified candidate metabolites in mechanistic tests and for use as analytical standards contributes to uncertainties about mechanism of action, toxicological profile and pharmacokinetic analysis in *in vivo* studies.^10^ Thus, there is a critical need for an effective and adaptable metabolite synthesis reactor that can produce sufficient quantities of metabolites to meet these important shortcomings in drug discovery pathways. Current methods for synthesising metabolites such as bacterial expression of enzymes, liver cell Incubations, liver microsomes and traditional organic synthesis suffer from various drawbacks such as extremely low yields,^11^ product formation inhibiting enzymatic activity^12^ and the necessity of a separation from a complex biological matrix.^13^

Microfluidic synthesis platforms have emerged as promising tools for the study of absorption, distribution, metabolism, and excretion with cell cultures of primary cells in 2D and 3D formats as well as tissues mimicking organs.^14^ Such organ-on-a-chip systems allow maintenance of cells, transport and removal of nutrients and reagents with precise control as well as control of environmental factors such as oxygen levels, temperature or shear stress. Organ-on-a-chip devices hold great promise in terms of studying drug metabolism and pharmacokinetics, especially if such devices are connected to allow modelling of multi-organ systems. However, despite this promise, organ-on-a-chip systems do not address the fundamental issues of allowing specific identification of the metabolites produced, and a direct assessment of the bioactivity/toxicity of each metabolite. There are several reports in the literature of the application of microfluidic technology to mimic drug metabolism in humans.^15^ These include utilising a microfluidic electrochemical cell simulating CYP450 oxidation and flow-through methods using PDMS membranes containing precision cut liver slices.^16,17^ However these studies either use non-specific chemical approaches to metabolising test compounds, such as electrochemical oxidation reactions,^18^ or lack specificity for producing a single metabolite by using complex mixtures of enzymes, such as microsomes or whole cells,^19,20^ or are aimed at the prediction of metabolism rather than any synthetic capacity.^21^ There are also some reports of the application of flow-through reactors to cytochromes P450 activity, however these are limited in scope and focus on the prediction of metabolism, rather than the generation of metabolites.^18–25^ Microfluidic flow synthesis platforms have demonstrated the ability to bypass challenges around batch synthesis. Enzyme immobilisation has been widely utilised throughout the literature.^26–28^ The need for complex separation required in batch processes can be bypassed *via* the use of immobilized enzyme systems.^29^ Inhibition can be minimised by the constant removal of products due to continuous flow through the microfluidic device.^30^ An immobilised enzyme usually has superior lifetimes and lower losses of activity and higher thermal and chemical stability compared to free enzymes. The covalent binding of enzymes provides high binding energies preventing enzyme leeching and provides a degree of reusability. Taken together, these reports on enzyme-based metabolic reactors coupled with the inherent capability of parallelisation of microfluidic flow chemistry, suggest that such an approach can address current challenges in drug metabolite synthesis.

**Fig. 1:**
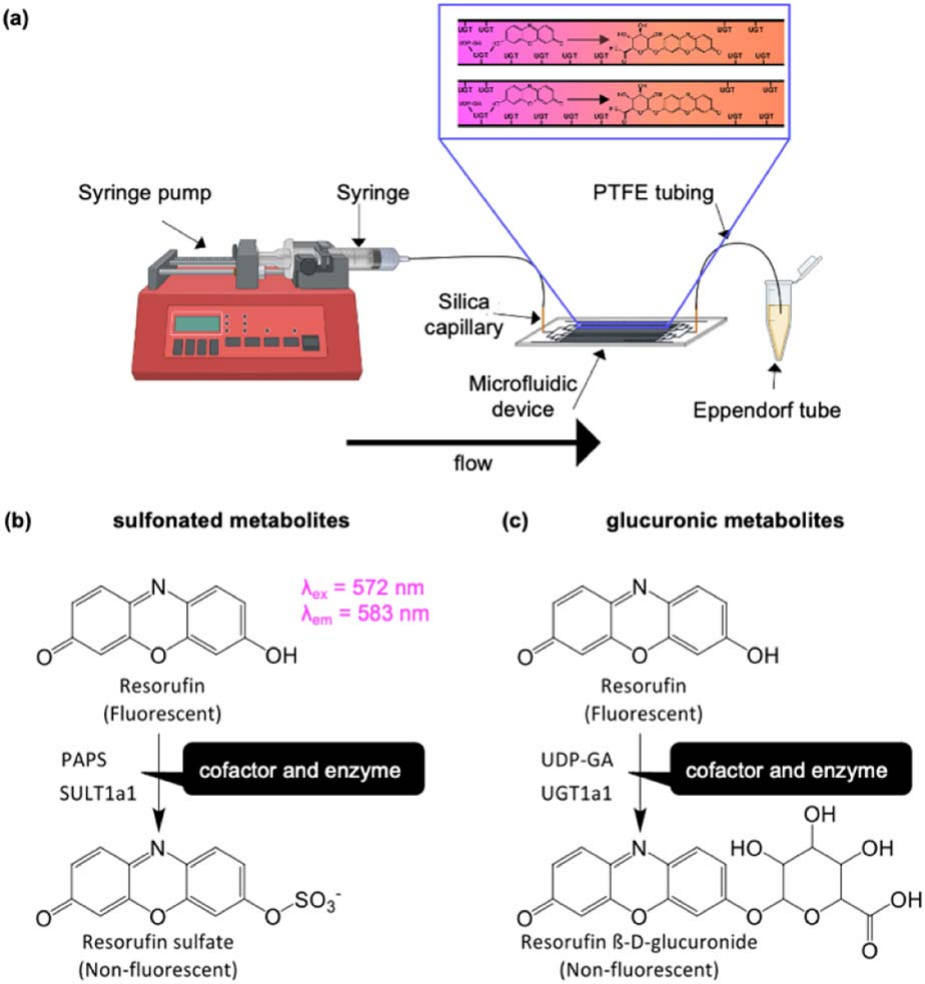
Concept of experimental design. (a) The setup featured a microfluidic device interfaced to a syringe pump *via* PTFE tubing and silica capillaries. Effluent was collected at the outlet. The inset shows the conversion of resorufin in the presence of UDP-GA cofactor with the enzyme UGT1a1 immobilised on the channel wall. (b) Reaction scheme for the metabolism of resorufin *via* sulfonation with SULT1a1 enzyme utilising PAPS as the co-factor. (c) Reaction scheme for the metabolism of resorufin *via* glucuronidation with UGT1a1 enzyme utilising UDP-GA as the co-factor.

Here we are addressing this task by investigating a microfluidic flow reactor with surface immobilised enzymes for conjugative metabolite synthesis. We demonstrate the concept with human SULT1a1 and UGT1a1 enzymes, two of the most common conjugative enzymes, using resorufin or *p*-nitrophenol as representative substrates for these enzyme reactors (Fig. 1).

The overall aim of this research was to develop a method which allows for the synthesis of metabolic products. A single enzyme was used in each device to allow the collection of a single product. In the present study, two compounds, resorufin and nitrophenol, were employed in order to determine metabolic conversion utilising this setup.

## Experimental

### Chemicals and reagents

UGT1a1 and SULT1a1 Supersomes were purchased from Corning (Netherlands). Sulfuric acid (96%), hydrogen peroxide (30%), sodium phosphate dibasic heptahydrate (98%), sodium phosphate monobasic monohydrate (98%), ethanol (95%), sodium hydroxide (98%), glutaraldehyde (25%), (3-aminopropyl)trimethoxysilane (97%), UDP-glucuronic acid (UDP-GA) (98%), 3′-phosphoadenosine-5′-phosphosulfate (PAPS, 60%), resorufin (95%), resorufin β-d-glucuronide (90%), *p*-nitrophenol (99%) and *p*-nitrophenyl sulfate (98%), LC-MS grade methanol, LC-MS grade water and triosephosphate isomerase, trifluoroacetic acid (TFA, 99%) were all obtained from Sigma-Aldrich (UK).

### Microfluidic device design and manufacture

Two chip designs were compared. Chip Design A featured a branched channel network with 16 parallel channels and Chip Design B featured a long single serpentine channel (ESI 1†). Briefly, in Chip Design A the parallel channels were each 50 mm long, 300 μm wide and etched to a depth of 30 μm. The surface area to volume ratio for this device was >5000 m^−1^. At a flow rate of 0.1 μL min^−1^ the residence time in the channels was 72 min. For Chip Design B, the serpentine was 667 mm long, 75 μm wide and etched to a depth of 30 μm with a surface area to volume ratio of 150 m^−1^. At a flow rate of 0.1 μL min^−1^ the residence time in the channel was 15 min.

**Fig. 2:**
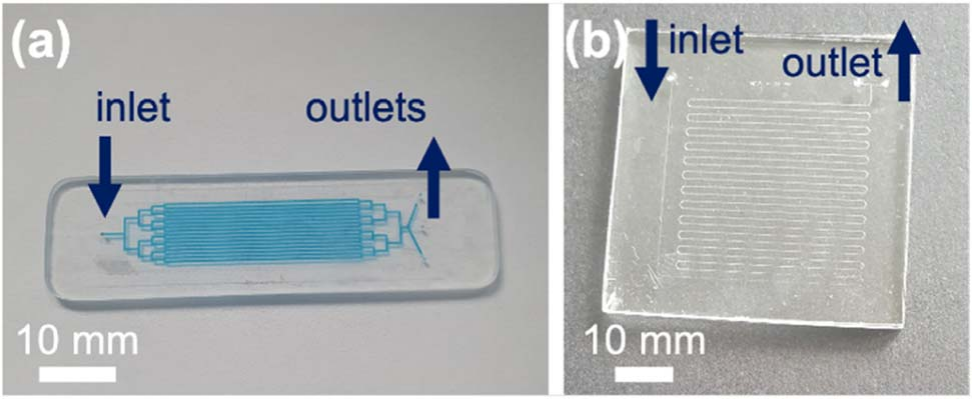
Photographs of glass microfluidic flow reactors. (a) Chip Design A with a branched structure leading to 16 parallel channels, each 50 mm long and 300 μm wide, etched to a depth of 30 μm. (b) Chip Design B featuring a 667 mm long winding serpentine channel of 75 μm width etched to a depth of 30 μm.

The microfluidic devices were fabricated in glass.^31,32^ Briefly, designs were printed on a photolithographic mask (JD Phototools) and transferred *via* photolithography onto glass (Schott B270, Tellic, USA) featuring a photoresist and chromium layer. Devices were then etched with hydrofluoric acid, access holes were drilled *via* a CNC machine (Datron) and devices were bonded through thermal fusion at 585 °C. Photographs of Device Design A and B are shown in Fig. 2. Devices were interfaced with PTFE tubing. Liquid was introduced under positive pressure with a Harvard Apparatus 11 Elite pump equipped with Terumo 1 mL plastic syringes. A photograph of the setup is shown in ESI 2.† The glass microfluidic devices were cleaned with piranha solution, *i.e.* 95% sulfuric acid and 30% hydrogen peroxide in a 3 : 1 ratio, for 2 h. The devices were then sonicated in water and allowed to dry. Silica capillaries were placed into the inlet and outlet holes and bound using epoxy resin 2 : 1.

### Immobilisation UGT1a1 and SULT1a1 on microfluidic devices

The reaction scheme for surface functionalisation is shown in ESI 3.† Following common functionalisation protocols, the glass surface was first silanised under introduction of an amino group with (3-aminopropyl)trimethoxysilane (APTMS)^33^ followed by glutaraldehyde^34^ and finally enzyme attachment.^35,36^ For both the parallel and the serpentine device, sodium hydroxide (0.1 M, 3 × 1 mL) was pumped through the chip by hand, followed by methanol (3 × 1 mL). To silanise the channel surface, a solution of (3-aminopropyl)trimethoxy silane (5% v/v in ethanol) was introduced into the flow cell and left for 5 min. This was washed out with methanol (3 × 1 mL). The device was then left in the oven at 60 °C for 1 h to dry the surfaces. Next, glutaraldehyde (5% v/v in 0.1 M phosphate buffer, pH 7.4) was pumped through the chip for one hour at 3 μL min^−1^. The final step was then to fill the channel with UGT1a1 (0.15 mg mL^−1^) or SULT1a1 (10 ng mL^−1^) solution. These were left in the fridge overnight. Metabolic conversions on the devices were studied with the substrate, *i.e.* resorufin/nitrophenol (100 μM in phosphate buffer), alongside the respective co-factor (UDP-GA for UGT1a1 and PAPS for SULT1a1), being pumped at varying flow rates utilising a NE-4000 syringe pump (0.1, 0.5 and 1 μL min^−1^).

### Assessment of UGT1a1 and SULT1a1 activity

Resorufin is a highly fluorescent molecule, with *λ*_ex_ = 572 nm and *λ*_em_ = 583 nm. However, upon conjugation to a glucuronic acid moiety it becomes non-fluorescent (see Fig. 1). This loss of fluorescence forms the basis for determining the extent of resorufin metabolism by our microfluidic reactors. Resorufin (100 μM) was pumped into the microfluidic device, effluent was collected, and the fluorescence intensity of the effluent was quantified using a calibration curve ranging from 0– to 100 μM. Flow rates of 0.1, 0.5 and 1 μL min^−1^ were investigated in Chip Design A or Chip Design B with immobilised UGT1a1 or SULT1a1. For experiments assessing different temperatures, devices were incubated at room temperature (20 ± 2 °C), 30 °C and 37 °C in a CO_2_ incubator (BB15, Thermo Scientific) with a run time of 2 h.

For comparisons to static enzyme reactions, 2 μL of SULT1a1 (10 ng mL^−1^) or UGT1a1 (0.15 mg mL^−1^) was added to a 10 μL mixture of 100 μM resorufin and co-factor (100 μM PAPS or 100 μM UDP-GA). They were left to incubate at 37 °C for 2 h. For all experiments, control reactions were carried out, where the cofactor, *i.e.* PAPS for sulfonation or UDP-GA for glucuronidation, was omitted from the reaction mixture. Devices were also prepared with immobilising the enzyme triose phosphate isomerase (0.15 mg mL^−1^) as an alternative enzyme, which was expected to yield no loss of fluorescence due to it having no specificity towards resorufin. These experiments were conducted to confirm whether the respective enzymes were necessary to obtain the loss of fluorescence.

### LC-MS-MS analysis of device effluents

Using the effluents from the SULT1a1 and UGT1a1 reactors, in the presence and absence of appropriate cofactor, resorufin glucuronide, *p*-nitrophenol glucuronide, resorufin sulfate, and *p*-nitrophenol sulfate were measured by LC-MS. SULT1a1 effluents were implemented directly and UGT1a1 effluents were extracted from the matrix using 10 μL C18 tips (Sigma-Aldrich). The C18 tip was wetted using 10 μL of 50% methanol in water twice, the tip was then equilibrated using 10 μL 0.1% TFA in water twice. Next, the sample was aspirated for 10 cycles and left in the tip for 10 min. This was followed by a rinse with 10 μL water twice and finally the sample was extracted from the tip using 10 μL of methanol.

Samples were analysed with a Shimadzu Nexera X2 series liquid chromatography system (Kyoto, Japan) connected to a Shimadzu Nexera X2 SIL-30AC coupled to a Shimadzu 8060 triple quadrupole mass spectrometer. Data acquisition and processing was performed by LabSolutions™ 5.93 software. The chromatographic separation was achieved on a Shim-pack GISS C18 column (50 mm × 2.1 mm, 1.9 μm) (Shimadzu). The mobile phase consisted of water for phase A and methanol for phase B, both containing 0.1% formic acid. The separation was carried out using a gradient method with mobile phase A : B set to 95% : 5% from 0.00 to 3.00 min, 25% : 75% from 10.00 to 20.00 min and then back to 95% : 5% from 22.00 to 25.00 min. The mass spectrometer was operated in negative ion mode. The nebulizer gas, collision gas, ion spray voltage and source temperature were set at 3 L min^−1^, 17 kPa, 2.32 kV, and 250 °C, respectively. A product ion scan in negative mode was used for product confirmation of both resorufin glucuronide and nitrophenyl sulfate with *m*/*z* of 388 and 218, respectively. Alongside this, multiple reaction mode (MRM) was used for further confirmation with selected transitions of 388 → 212 and 218 → 138 *m*/*z*, respectively.

### Statistical analysis

All statistical analysis was performed using Graphpad Prism version 8. Normality of data was assessed by Shapiro–Wilks test. Parametric data was assessed by One-way ANOVA with Bonferroni post hoc test.

## Results

### Sulfonation of resorufin in SULT1a1 reactors

**Fig. 3:**
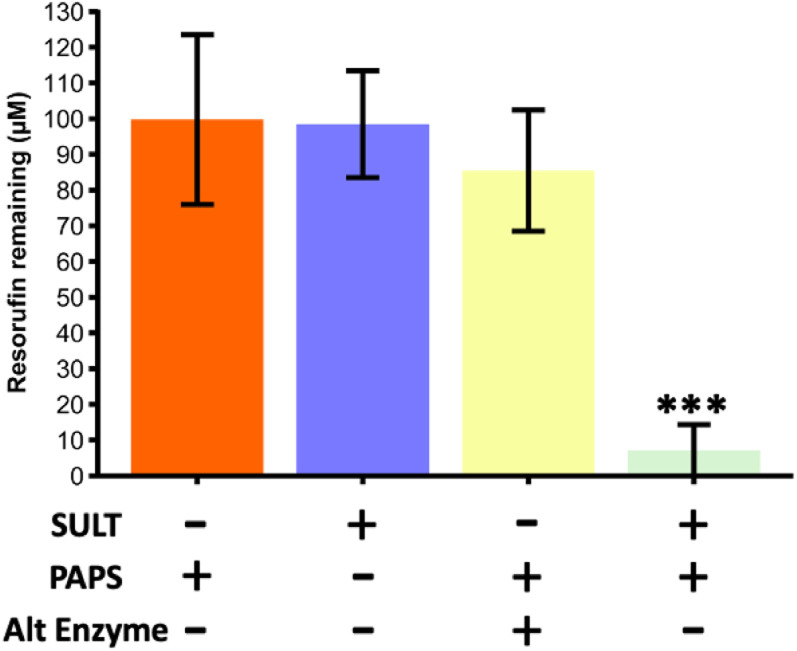
Resorufin remaining from a starting concentration of 100 μM for a series of reaction runs through the microfluidic device with parallel channels (Chip Design A). Only during the true run with the SULT1a1 enzyme immobilised on the channel wall and the correct co-factor (PAPS) present, was the fluorescence of the resorufin significantly reducing, indicating its conversion. Runs with just the cofactor, with just the enzyme and with the co-factor and an alternative enzyme (triosephosphate isomerase) did not yield in a significant reduction of fluorescence. The true run was found to be significantly lower than all three of the blanks compared (one way ANOVA with Bonferroni corrections, *** denotes significance (*p* < 0.001, *n* = 3)).

To study the metabolic reaction of sulfonation, SULT1a1 enzyme was used together with PAPS co-factor.^37^ Initial experiments were carried out in Chip Design A (parallel channels) with a flow rate of 0.5 μL min^−1^ at room temperature (20 ± 2 °C). In order to assess SULT1a1 activity, the loss of resorufin fluorescence was monitored, since resorufin is highly fluorescent and its conjugated derivatives are not. A series of experiments was carried out, with a true run featuring the SULT1a1 enzyme and PAPS co-factor, as well as control experiments with the enzyme or co-factor missing, and also with an alternative enzyme (triosephosphate isomerase) in the presence of the co-factor (Fig. 3). The experiments showed that resorufin fluorescence was only reduced in the microfluidic flow reactor with the SULT1a1 enzyme immobilised on the channel walls and only in the presence of the PAPS co-factor. When either the PAPS co-factor or the SULT1a1 enzyme were absent, resorufin fluorescence was unchanged. To assess if resorufin might non-specifically interact with proteins attached to the microfluidic device, which could also cause a reduction in resorufin detection in the device effluent, we lined the device with the enzyme triosephosphate isomerase. This enzyme is of similar molecular weight to SULT1a1 but should have no activity towards resorufin. As can be seen in Fig. 3, under these conditions, resorufin fluorescence was unchanged, demonstrating that the observed loss of fluorescence in the SULT1a1 devices in the presence of PAPS is a specific effect, and indicative of resorufin metabolism.

### Comparison of parallel *versus* serpentine channel designs

**Fig. 4:**
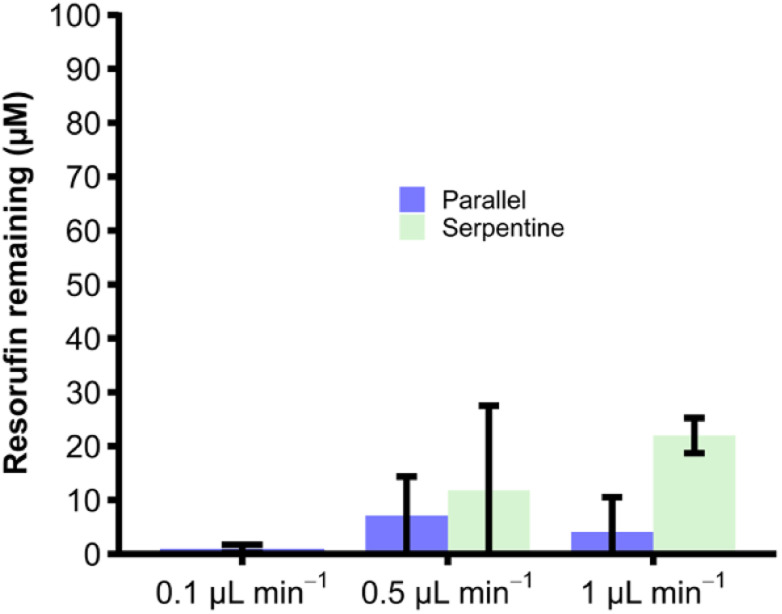
Amount of resorufin remaining at three different flow rates for sulfation via SULT1a1. Mean concentration of resorufin remaining with ± SD from a starting concentration of 100 μM upon metabolism *via* SULT1a1 immobilised in the two types of flow devices, the parallel channel reactor (Chip Design A) and the serpentine channel flow reactor (Chip Design B). Experiments were performed at three different flow rates, *i.e.* 0.1 μL min^−1^, 0.5 μL min^−1^ and 1 μL min^−1^. No significant difference found between any of the measured flow rates (one way ANOVA with Bonferroni corrections, (*p* > 0.05, *n* = 3)).

Two different microfluidic channel designs were compared, a design with 16 parallel channels (Chip Design A) and a design with a long winding single serpentine channel (Chip Design B) (see Fig. 2) The resorufin concentration remaining in the eluent at different flow rates ranging from 0.1 μL to 1 μL min^−1^ was assessed, with loss of fluorescence taken as indicating conjugative metabolism (Fig. 4). As can be seen, within both microfluidic devices the resorufin concentration was decreased substantially from its starting concentration of 100 μM. However, the data appeared to be more consistent for the parallel channel devices. At all flow rates no significant difference was found for both parallel ([resorufin] 0.7 ± 0.7 μM, 6.0 ± 6.9 μM and 4.2 ± 5.5 μM resorufin remaining at 0.1, 0.5 and 1 μL min^−1^, respectively) and serpentine devices ([resorufin] 11.8 ± 15.7 μM and 22.0 ± 3.3 μM at 0.5 and 1 μL min^−1^, respectively). Due to its superior product formation, the parallel channel reactor with the larger surface to volume ratio was utilised for all subsequent experiments.

### Effect of incubation temperature on enzyme activity

**Fig. 5:**
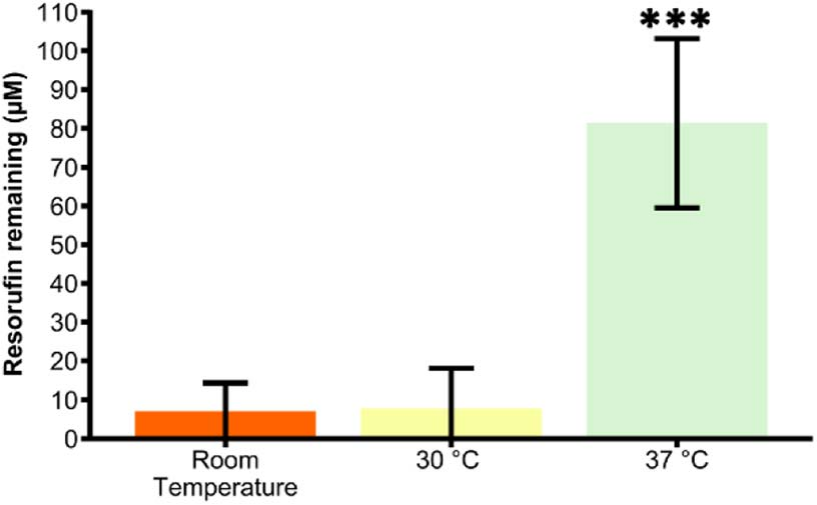
Enzymatic conversion at three different temperatures for SULT1a1 metabolism of resorufin. Concentration of resorufin remaining upon metabolism *via* flowthrough of SULT1a1 immobilised device against three different temperatures. 37 °C significantly higher than the two other tested temperatures (one-way ANOVA with Bonferroni corrections, *** denotes significance (*p* < 0.001, *n* = 3)).

Initial experiments were undertaken at room temperature (20 ± 2 °C) for ease. Next we assessed the effect on reactor activity when increasing temperature to 30 °C and to 37 °C, the latter the optimum temperature for this enzyme as provided by the supplier. Results for the remaining resorufin fluorescence from a starting concentration of 100 μM are shown in Fig. 5. At room temperature and 30 °C, the resorufin concentration in the effluent was measured at = 7.1 ± 7.2 μM and 7.8 ± 10.2 μM, respectively. There was no significant difference in loss of resorufin for these two conditions (*p* > 0.05, One-way ANOVA with Bonferroni post hoc test). In contrast, at 37 °C resorufin concentration was found to only be reduced from 100 M to 81.3 ± 21.8 μM, which is significantly higher than the values obtained for either room temperature or 30 °C (*p* < 0.001, one-way ANOVA with Bonferroni post hoc test). All subsequent experiments were thus carried out at room temperature as immobilising the enzyme likely had an effect on the optimum reaction temperature.

### Comparison of flow-through reactors with static incubations

**Fig. 6:**
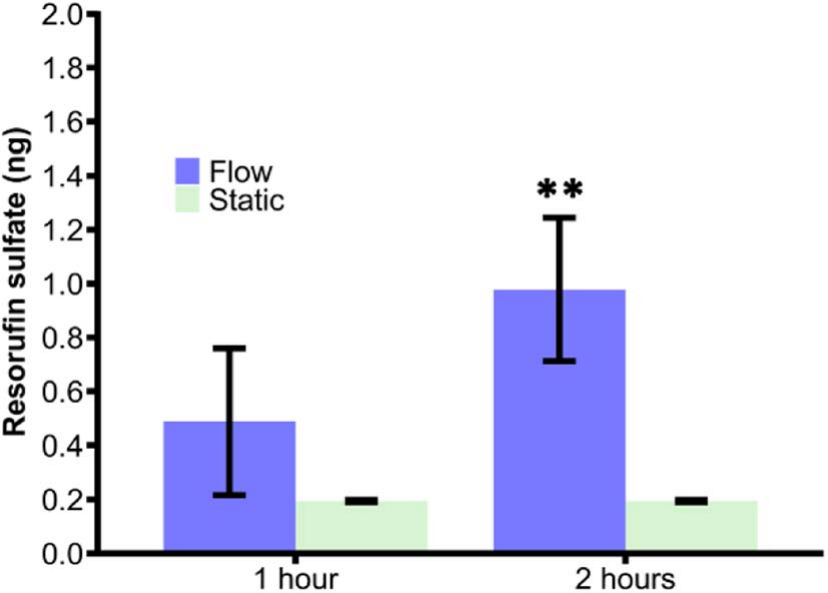
Comparison of resorufin sulfate formed in both a flow and static method over 1 h and 2 h via SULT1a1. The concentration of resorufin remaining upon metabolism in the flow device with SULT1a1 immobilised on the channels walls for a 2 h run time were significantly higher than all three of the other times/methods compared (one-way ANOVA with Bonferroni corrections, ** denotes significance (*p* < 0.001, *n* = 3)).

To demonstrate that the microfluidic reactors were a viable approach to synthesise drug metabolites, the product yield, measured by loss of resorufin fluorescence, was compared to that of SULT1a1 reactions in a static system (Fig. 6). Static reactions were run for 1 h and 2 h and compared those in the flow reactor at 0.1 μL min^−1^ (Fig. 6). The two methods did not yield a significant difference in the amount of product formed over the 1 h period, 0.19 ± 0.00 μg for static incubation *versus* 0.49 ± 0.27 μg for the flow-through approach. Allowing the reactions to continue for 2 h, significantly more product was formed in the flow reactor (0.97 ± 0.26 μg) than *via* the static incubation which indeed yielded no further product formation. Although there is more variation in the flow-through device, consistently more product was formed over a longer period of time.

### Confirmation of sulfonation activity using LC-TQMS

Sulfonation activity of the SULT1a1 enzyme was investigated *via* liquid chromatography triple quadrupole mass spectrometry (LC-TQMS) for both resorufin as well as an alternative substrate, *i.e. p*-nitrophenol. The identification of resorufin and resorufin metabolites using LC-TQMS showed that the resorufin stock contained a resorufin sulfate-like contaminant. Both retention time and *m*/*z* were predicted as resorufin sulfate (data not shown). Therefore *p*-nitrophenol, a classical SULT1a1 substrate, was used as an alternative substrate for these experiments.

**Fig. 7:**
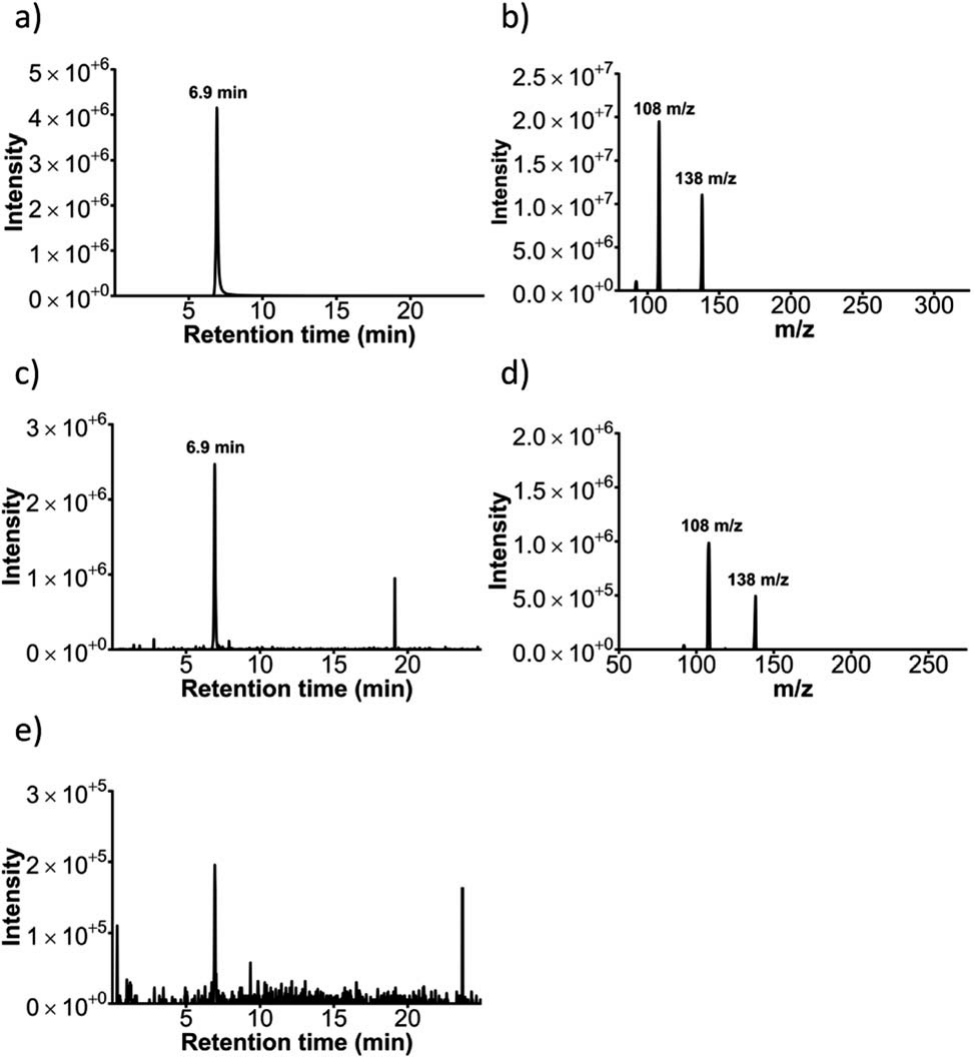
Confirmation of sulfonation via LC-TQMS. (a) Chromatogram of a nitrophenol sulfate standard with an ion scan at 218 *m*/*z* showing a peak for nitrophenyl sulfate at 6.9 min and (b) its respective mass spectrum showing the expected parent ion (138 *m*/*z*) for nitrophenol and its frequently observed fragment ion. (c) Chromatogram of effluent from a run through with *p*-nitrophenol being pumped through the flow reactor with SULT1a1 enzyme immobilised and the PAPS co-factor present, showing the peak for the nitrophenol sulfate product at 218 *m*/*z* and (d) its respective mass spectrum. (e) Chromatogram with a product ion scan at 218 *m*/*z* of effluent when pumping *p*-nitrophenol through a reactor with SULT1a1 enzyme immobilised but without the addition of the PAPS co-factor.

A chromatogram of a nitrophenol-sulfate standard solution with *m*/*z* = 218 shows the retention time as 6.9 min (Fig. 7a). Its respective mass spectrum shows a loss of *m*/*z* = 80 (218 → 138), which is characteristic for sulfate conjugates (Fig. 7b).^38^ Effluent from the SULT1a1 microfluidic reactors incubated with PAPS and *p*-nitrophenol (2 h, 0.5 μL min^−1^) contained a metabolite identified as nitrophenol-sulfate, based on *m*/*z* = 218 and the retention time of 6.9 min (Fig. 7c). Both this *m*/*z* and retention time matched those of an analytical standard of nitrophenol-sulfate. The MRM (218 → 138), representing a loss of *m*/*z* = 80, which is characteristic for sulfate conjugates, was also observed in the SULT1a1 reactor effluents (Fig. 7d), and were in agreement with those observed for the nitrophenol-sulfate standard. For SULT1a1 reactors incubated with *p*-nitrophenol without the addition of the PAPS co-factor (Fig. 7e), the *m*/*z* = 218 ions at 6.9 min retention time were minimally detected in either single ion monitoring (at *m*/*z* = 218) or using the MRM 218 → 138 method. Taken together, these data demonstrate that the SULT1a1 reactors yield nitrophenol-sulfate, and this metabolism specifically occurs only when the SULT1a1 enzyme and co-factor are present.

### Glucuronidation of resorufin with UGT1a1

**Fig. 8:**
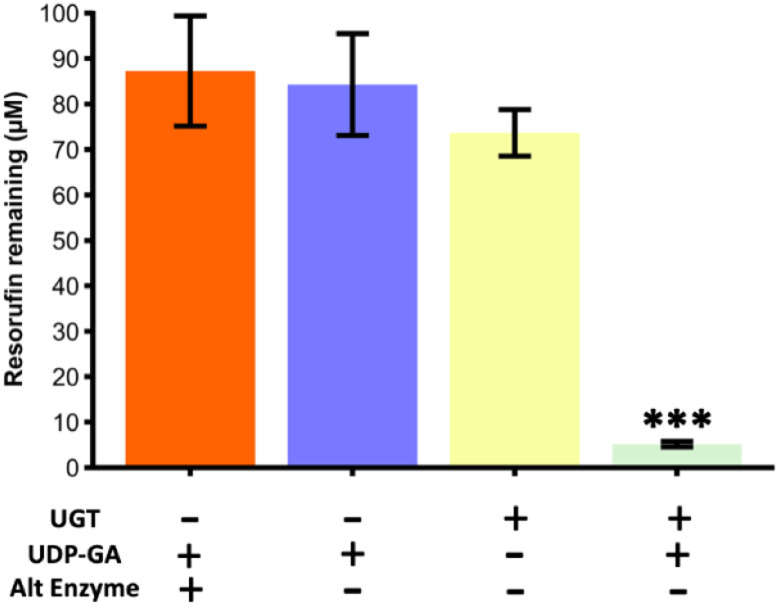
Glucuronidation of resorufin from a starting concentration of 100 μM for a series of reaction runs through the microfluidic device with parallel channels (Chip Design A). Only during the true run with the UTG enzyme immobilised on the channel wall and the correct co-factor (UDP-GA) present, was the fluorescence of the resorufin significantly reduced, indicating its conversion. Runs with just the cofactor, with just the enzyme and with the co-factor and an alternative enzyme (triosephosphate isomerase) did not yield in a significant reduction of fluorescence. The true run was found to be significantly lower than all three of the blanks compared (one-way ANOVA with Bonferroni corrections, *** denotes significance (*p* < 0.001, *n* = 3)).

As a second type of enzymatic reaction, the glucuronidation of resorufin with UGT1a1 enzyme and UDP-GA cofactor was studied (see Fig. 1c). Experiments were carried out in Chip Design A (parallel channel design) at a flow rate of 0.1 μL min^−1^ at room temperature. As for the SULT1a1 reactor, the glucuronidation activity was measured by loss of resorufin fluorescence. A series of experiments was carried out, with a true run featuring the UTG enzyme and UDP-GA co-factor, as well as control experiments with the enzyme or co-factor missing, and with an alternative enzyme (triosephosphate isomerase) in the presence of the co-factor (Fig. 8). The results showed that resorufin fluorescence was only reduced when the UGT1a1 enzyme was immobilised on the channel walls, and only in the presence of the UDP-glucuronic acid (UDP-GA) co-factor. When either the UDP-GA co-factor or the UGT1a1 enzyme were absent, resorufin fluorescence was unchanged. When triose phosphate isomerase was immobilised instead of UGT1a1, no reduction in resorufin fluorescence was observed, demonstrating that the observed loss of fluorescence in the UGT1a1 devices in the presence of UDP-GA is a specific effect, and indicative of resorufin metabolism.

### UGT1a1 flow reactors compared to static incubation

**Fig. 9:**
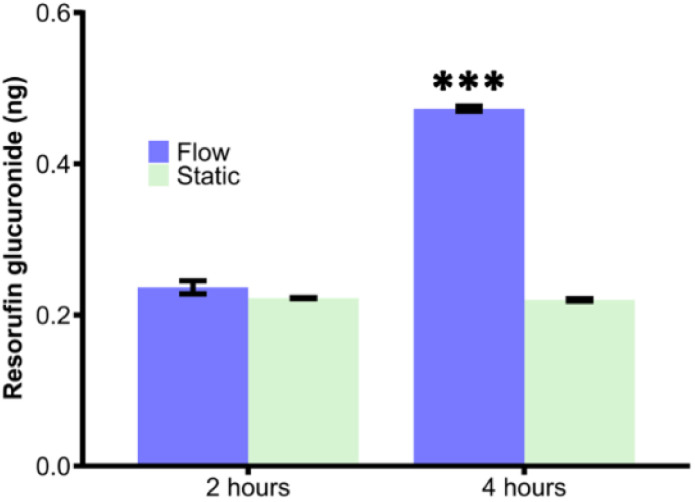
Comparison of resorufin glucuronide formed via UGT1a1 under both static and flow-through conditions over 2 h and 4 h. Resorufin glucuronide formed upon metabolism *via* the flow-through device with UGT1a1 immobilized compared to resorufin glucuronide formed under static incubation. The obtained resorufin in the 4 h flow conditions was significantly higher than the 2 h run and also the static runs (one-way ANOVA with Bonferroni corrections, *** denotes significance (*p* < 0.001, *n* = 3)).

Reactions of UGT1a1 in a microfuge tube at static conditions as per manufacturer's instruction and in the parallel channel device at 0.1 μL min^−1^ were run for 2 h and 4 h, with the amount of resorufin converted (from micromolar to ng) determined from the loss of resorufin fluorescence compared to the starting concentration of resorufin for these reactions (Fig. 9). It was observed that over 2 h, there was no significant difference between the flow-reaction and static incubation (*p* > 0.05, one-way ANOVA with Bonferroni post hoc test). However the flow-through reactors continued to metabolise resorufin, resulting in a significant increase in the amount of product yielded compared with static reactions at the 4 h time point (*p* < 0.001, one-way ANOVA with Bonferroni post hoc test).

### Confirmation of glucuronidation activity *via* LC-TQMS

**Fig. 10:**
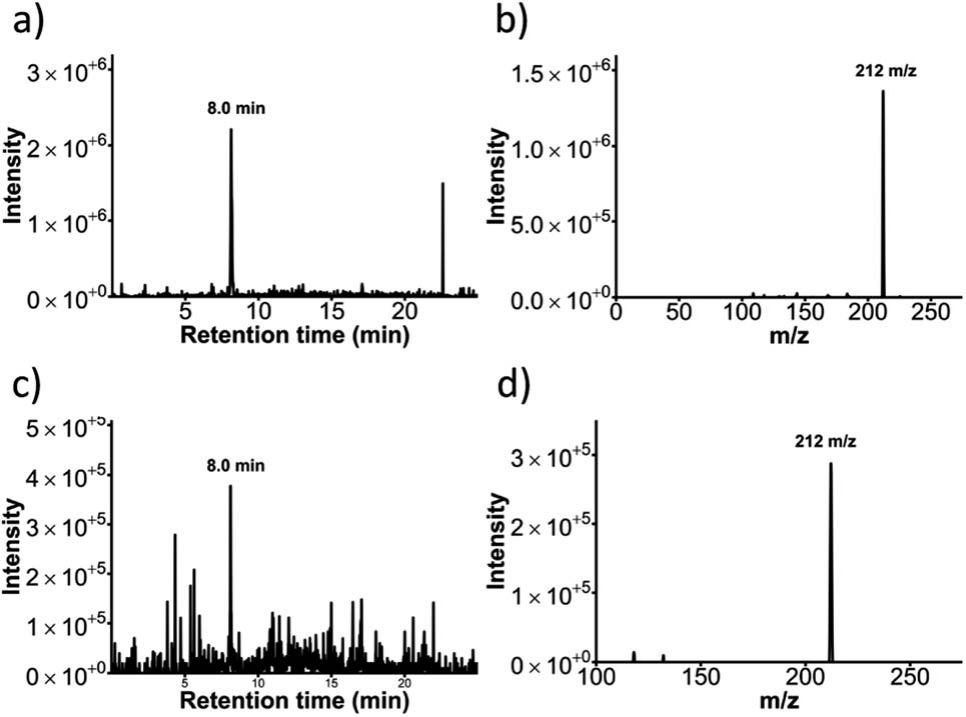
Confirmation of glucuronidation via LC-TQMS (a) Chromatogram of a resorufin glucuronide standard with an ion scan at 388 *m*/*z* showing a peak for resorufin glucuronide at 8.0 min and (b) its respective mass spectrum showing a peak at *m*/*z* = 212. (c) Chromatogram of an effluent from a run with resorufin glucuronide being pumped through the flow reactor with UGT enzyme immobilised and the UDP-GA co-factor present, showing the peak for the resorufin glucuronide product at 388 *m*/*z* and (d) its respective mass spectrum.

Glucuronidation activity of the UGT1a1 enzyme was investigated *via* liquid chromatography triple quadrupole mass spectrometry (LC-TQMS) for resorufin. A chromatogram of a resorufin-glucuronide standard solution with *m*/*z* = 388 shows the retention time as 8.0 min (Fig. 10a). Its respective mass spectrum shows fragment *m*/*z* = 212, representing a loss of *m*/*z* = 176, which is characteristic for glucuronide conjugates.^39^ Effluent collected from the UGT1a1 flow reactor incubated with both UDP-GA and resorufin (2 h, 0.1 μL min^−1^) contained a metabolite identified as resorufin-glucuronide, based on *m*/*z* = 388 and a retention time of 8.0 min (Fig. 10c). Both the *m*/*z* and the retention matched those of the analytical standard for resorufin d-glucuronide. The MRM (388 → 212), representing a loss of *m*/*z* = 176 was also observed in the effluent of the UGT1a1 reactor when co-factor was present (Fig. 10d) and were, again, in agreement with those observed for the resorufin-glucuronide standard. For UGT1a1 reactors incubated with resorufin without the addition of the UDP-GA co-factor the *m*/*z* = 212 ions at 8.0 min retention time were minimally detected in either single ion monitoring (at *m*/*z* = 388) or using the MRM 388 → 212 methods.

## Discussion

The overarching aim of this research was to try and address the current challenges in synthesising metabolites of drugs, which are needed for *in vitro* compound testing. The current lack of ability to readily access drug metabolites is a widespread limitation for *in vitro* mechanistic research.^40^ The research presented in this paper applies immobilized-enzyme flow-reactors, using human drug metabolism enzymes, as a synthesis reactor for metabolites. We demonstrated that for sulfation and glucuronidation enzymes, sulfate and glucuronide metabolites of resorufin and *p*-nitrophenol, two well described substrates for these conjugative pathways, can be generated in nanogram quantities in a few hours, without the need for specialist equipment or complex sample preparation and purification. These data suggest that the use of this microfluidic approach has a role in drug metabolite synthesis.

The method described in the present study offers a relatively simple route to the production of metabolites and could be adopted quickly by research groups with limited microfluidic experience due to its ease of setup and use. These data suggest that the use of this microfluidics approach has a role in drug metabolite synthesis. Whilst the here presented microfluidic device was fabricated in house with specialist equipment, flow chemistry devices can be readily purchased from a range of suppliers and the protocols employed here can be readily adapted to such systems. The glass microfluidic devices used here can be cleaned with piranha and heat treatment and reused for a period of several years.

It should be noted that other drug metabolism enzymes have been previously reported in microfluidic devices,^16,41,42^ as have the use of more complex systems such as microsomal fractions, hepatocyte cells, and single cell organisms. However, it is important to acknowledge that these systems have primarily been aimed at predicting which drug metabolites are being produced, rather than being used as a synthetic pathway to yield useable quantities of metabolites. Additionally, the integration of multiple different enzymes, *e.g.*, microsomal fractions,^43,44^ whole cells,^45,46^ cell lysates,^47,48^ can result in the production of several metabolites in the same reaction, meaning that purification of individual metabolites would be required to use these devices as a synthetic approach. It should also be acknowledged that there are several other methods available for synthesising human drug metabolites, including bacterial cultures,^49^ purification from health volunteer plasma,^50^ and traditional organic chemistry approaches.^51^ However, these other routes of synthesis often require either specialist facilities, specialist expertise, or access to healthy volunteers and preparative purification equipment. This study has demonstrated that using a microfluidics approach, specific metabolites can be produced, with a significantly increased yield compared to static enzyme incubations. There are also several published examples of enzyme-immobilised microfluidic reactors used for synthesis functions, highlighting the scalability of this approach.

There are commercial flow synthesis systems that demonstrate scaling up and scaling out of microfluidic flow chemistry, including the Plantrix® MR555 device (Chemtrix B.V.) and the desktop chemical plant system from IMT-Taiwan that runs microfluidic chips in series and multiple parallels to achieve high throughputs. Another example of scaling out is the parallelisation of 180 devices for emulsion formation.^52^

Over the running time of 4 h, there was no loss of activity observed. Further stability studies could be undertaken to determine the longevity of these devices.

Limitations of this study include the use of a loss of fluorescence assay which may not be fully representative of product formed. However, product formation was confirmed *via* LC-MS demonstrating these devices are yielding metabolites. Also with these initial proof of concept experiments relatively simple substrates have been employed. Further studies will also include more directly pharmaceutically relevant compounds and drugs. Finally, kinetics calculations have not been included in this study as quantitation of the immobilised enzyme has not been conducted due to the focus being that of a synthetic method. A further study to determine the catalytic properties of this device would yield further clarity on the devices' functionality.

Taken together, the findings in this paper and the scalability of microfluidic devices, strongly suggest that this approach to the synthesis of metabolites holds great promise to address an important barrier in pharmacological research.

The focus on this research has been on determining capability with regards to forming metabolic products. This led to the initial determination using test molecules chosen based on their inherent drug-like features. *Para*-nitrophenol is a precursor used in the synthesis of several drugs, *e.g.* paracetamol and fungicides, and thus has inherent drug-like qualities. With regards to resorufin, although it is best known as a cellular fluorescent probe, it is well associated with conjugative reactivities, and thus representative of a number of xenobiotics, *e.g.* polyaromatic hydrocarbons, and endogenous, *e.g.* steroids, substrates of both UGT and SULT enzymes.

Stability studies of these reactors would be needed to gauge if the process could run for even a few days with constant product formation. The enzyme loading after immobilisation was not quantified but product formation confirms its presence. Evaluating enzyme loading would help to evaluate the likelihood of long-term performance and scalability.

## Conclusions

We have demonstrated a method that allows for the synthesis of both sulfate and glucuronide conjugated metabolites using a covalently linked enzyme within a glass microfluidic device. This method provides high overall yields of 0.97 ± 0.26 μg over 2 h for the sulfated molecules and of 0.47 ± 0.003 μg over 4 h for glucuronide conjugated products, whilst also bypassing the common issue of a separation from a complex biological matrix. This shows that the here proposed method is feasible for the synthesis of naturally circulating metabolites and, upon scaling up, will be viable for the synthesis of standards that can be used for further studies.

## Author contributions

Funding acquisition and conceptualisation by HSJ and NP. Experiments were carried out by BD, with data analysis supported by LM, NP and HSJ. The manuscript was drafted by HSJ and edited by BD, LM and NP.

## Conflicts of interest

There are no conflicts to declare.

## Supplementary Material

RA-013-D3RA03742H-s001
