# Supplementary material for: Immobilised-enzyme microreactors for the identification and synthesis of conjugated drug metabolites
Source: RSC Adv. 2023 Sep 18;13(40):27696–704. doi: 10.1039/d3ra03742h (PMC10506384; doi:10.1039/d3ra03742h)
Supplement: RA-013-D3RA03742H-s001 [file RA-013-D3RA03742H-s001.pdf]

Electronic Supplementary Material (ESI) for RSC Advances.
This journal is © The Royal Society of Chemistry 2023

Electronic Supplementary Information

Immobilised-enzyme microreactors for the identification and synthesis of conjugated drug metabolite

Bradley Doyle[1], Leigh A. Madden[2], Nicole Pamme*[1,3], Huw S. Jones[4*]

[1]School of Natural Sciences, University of Hull, HU6 7RX, UK

[2] Centre for Biomedicine, University of Hull, HU6 7RX, UK

[3]Department of Materials and Environmental Chemistry, Stockholm University, 106-91 Stockholm, Sweden

[4]Institute of Cancer Therapeutics, University of Bradford, BD7 1DP, UK

*Corresponding author (h.s.jones@bradford.ac.uk)

*Corresponding author (h.s.jones@bradford.ac.uk)

ESI 1 – Microfluidic Chip designs

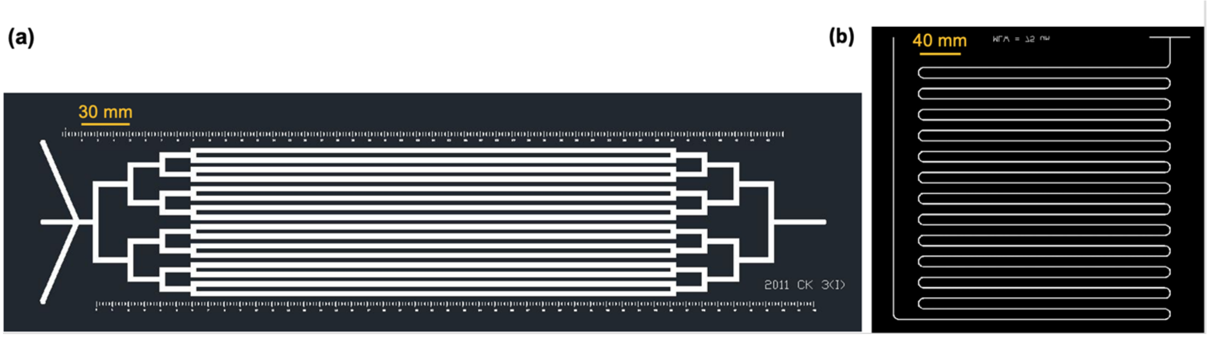

Figure S1: **AutoCAD drawing of channel designs.** (a) Chip Design A (parallel channel network) and (b) Chip Design B (serpentine channel).

Table S1: Specifications for parallel and serpentine chip designs.

	**Chip Design A** (parallel channels)	**Chip Design B** (serpentine channel)
Channel length (mm)	50	667
Channel width (μm)	300	75
Channel etch (μm)	30	30
Retention time (min) at a flow rate of 0.1 μL min^{-1} assuming width at half depth	76	18
Surface area to volume ratio (m^{-1})	5400	150

ESI 2 - Experimental Setup

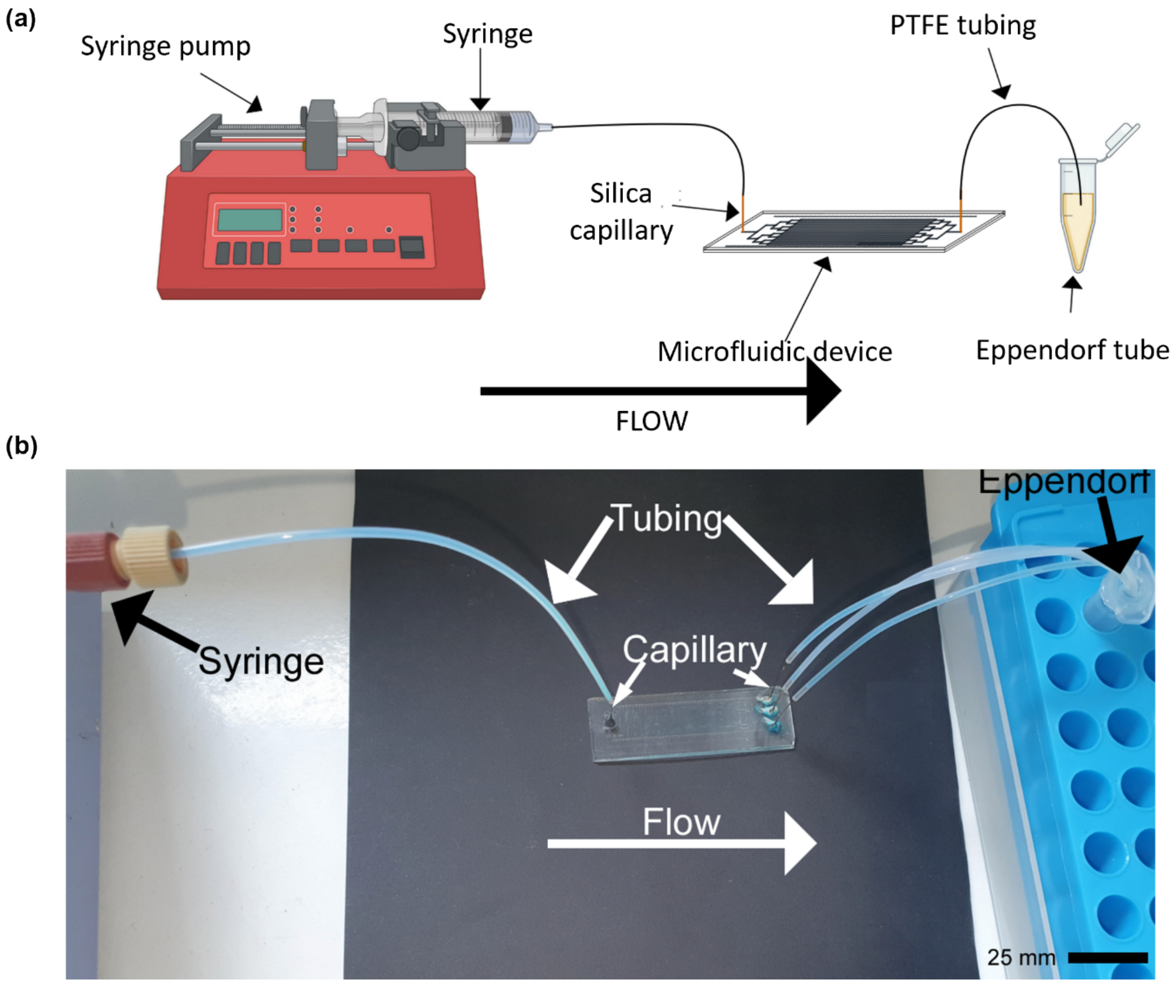

Figure S2: (a) Schematic drawing of the setup featuring the microfluidic chip interfaced to a syringe pump operated under positive pressure via PTFE tubing. A short piece of a silicon capillary was glued onto the microfluidic device. Effluent was collected in Eppendorf tubes. (b) Photograph of the setup.

ESI 3 - Surface Immobilisation

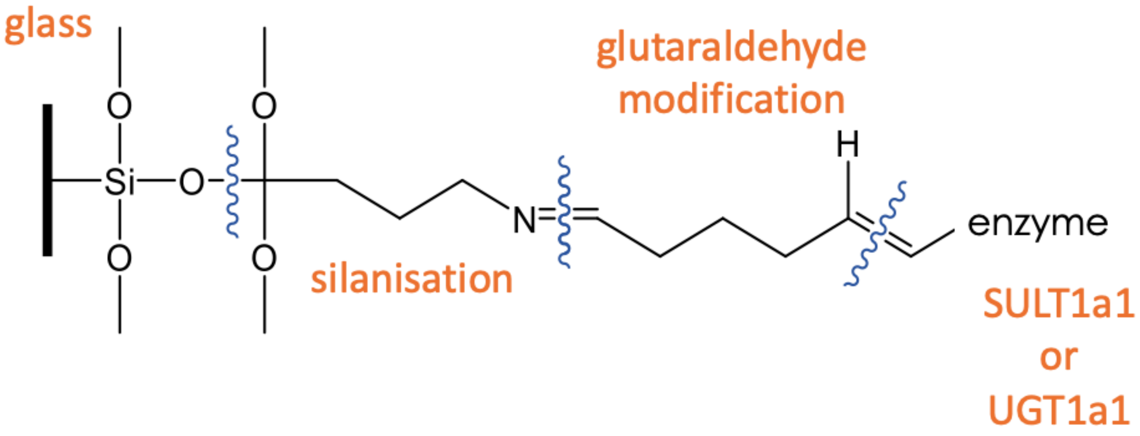

Figure S3: Surface immobilisation of enzymes on glass channels. Following flushing with sodium hydroxide and methanol, 3-(Aminopropyl) trimethoxy silane (5% v/v in ethanol) was introduced and left to incubate for 5 min. This was washed out with methanol and left to dry at 60 °C for an hour. Next glutaraldehyde (5% v/v in 0.1 M phosphate buffer, pH 7.4) was pumped for 1 h at 3 µL min^{-1}. Finally, the enzyme solutions, *i.e.* SULT1a1 (10 ng mL^{-1}) or UGT1a1 (0.15 mg mL^{-1}) were introduced and left to incubate in the fridge overnight.
